# Can Chimpanzee Biology Highlight Human Origin and Evolution?

**DOI:** 10.5041/RMMJ.10009

**Published:** 2010-07-02

**Authors:** Itai Roffman, Eviatar Nevo

**Affiliations:** International Graduate Center of Evolution, Institute of Evolution, University of Haifa, Mount Carmel, Haifa, Israel

**Keywords:** Hominin, evolution, *Homo*, chimpanzee, bonobo, common origins

## Abstract

The closest living relatives of humans are their chimpanzee/bonobo (*Pan*) sister species, members of the same subfamily “Homininae”. This classification is supported by over 50 years of research in the fields of chimpanzee cultural diversity, language competency, genomics, anatomy, high cognition, psychology, society, self-consciousness and relation to others, tool use/production, as well as *Homo* level emotions, symbolic competency, memory recollection, complex multifaceted problem-solving capabilities, and interspecies communication. Language competence and symbolism can be continuously bridged from chimpanzee to man. Emotions, intercommunity aggression, body language, gestures, facial expressions, and vocalization of intonations seem to parallel between the sister taxa *Homo* and *Pan*. The shared suite of traits between *Pan* and *Homo* genus demonstrated in this article integrates old and new information on human–chimpanzee evolution, bilateral informational and cross-cultural exchange, promoting the urgent need for *Pan* cultures in the wild to be protected, as they are part of the cultural heritage of mankind. Also, we suggest that bonobos, *Pan paniscus,* based on shared traits with *Australopithecus*, need to be included in Australopithecine’s subgenus, and may even represent living-fossil Australopithecines. Unfolding bonobo and chimpanzee biology highlights our common genetic and cultural evolutionary origins.

## INTRODUCTION

### HUMAN–CHIMPANZEE SISTER SPECIES STATUS

Can human nature be better comprehended by unfolding chimpanzee nature? Here, Einstein’s idea is of substantial significance: “The most incomprehensible thing about the world is that it is comprehensible.” We will try to substantiate our conviction that chimpanzees share much in their biology (morphology, anatomy, physiology, behavior, genetics, genomics, and culture) with humans, and that this understanding will not only reinforce the idea that chimpanzees are closely related biologically to humans, but also that humans share biological roots with chimpanzees, which are expressed in our current biological profiles. Dobzhansky^1^ defined Man as the “most mysterious of all experiences. This is why art and science strive to make him comprehensible*”*. Darwin reinforced the idea that man is related to all life-forms and is not a separate creation: “We must acknowledge, as it seems to me, that man with all his noble qualities, with his god-like intellect which has penetrated into the movements … of the solar system. Man still bears in his bodily frame the indelible stamp of his lowly origin”.^2^ “Light”, said Darwin “will be thrown on the origin of man and his history”^3^ (see supplementary material, sections 1., 1.1.).

**Figure 1:**
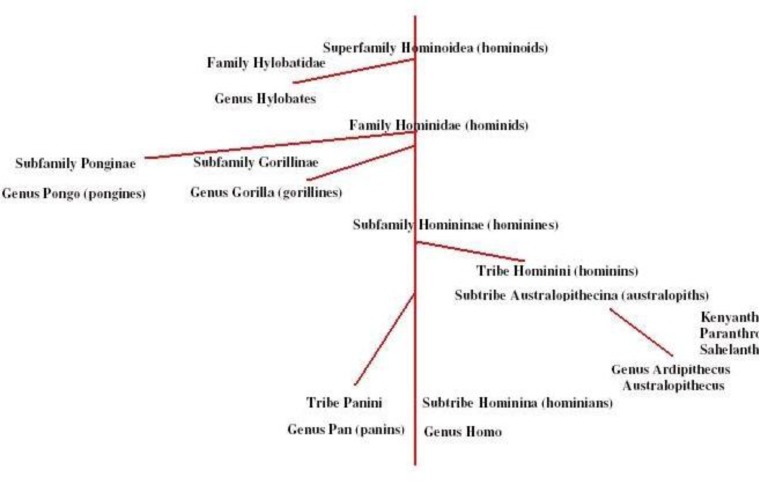
phylogenetic tree of *Pan/Homo* relations

Since evolution is on-going, man continues to evolve and so do chimpanzees; both share much in their genotypic and phenotypic heritage, and it is our goal to unfold this biological link for understanding man, chimpanzees, and their mutual evolutionary relationships. The closest living relatives of humans are their chimpanzee/bonobo sister species, members of the same subfamily “Homininae”, which include human lineage (*Homo*) and chimpanzees/bonobos (*Pan*) as sister species at the level of tribes together with Australopithecines^4^–^7^ (Figure 1).

However, time and again, claims are made that man is uniquely separated from other life-forms because of the following suite of characteristics: mental faculties, which is an impassable barrier from all animals by displaying progressive improvement, use of tools or fire, domestication, property, language, conscience, comprehension, abstraction, ideas, expressing gratitude, mystery and endowed with a belief in God (for elaboration see supplementary material, sections 1., 1.1., 2.1.). In this essay on chimpanzees and bonobos, we summarize current, old, and new information related to these major seemingly differentiated “human traits”. We demonstrate that all of these traits exist either developed or as seeds in our sibling species: the bonobos (*Pan paniscus*) and chimpanzees (*Pan troglodytes*).

## REDEFINING CHIMPANZEE–HUMAN RELATIONS

“‘Ape-grade’ problem-solving is not sharply distinguished from the early hominids. The key word in the evolution of human cognition and language is representation[al] symbolic art – the clearest symbol [of] abstract notion”.^8^ In this regard, according to A. Ronen (personal communication, 2009), “informational exchange” is a unique *Homo* trait, and, in this article, we provide preliminary evidence suggesting that *Pan* (chimpanzees and bonobos) fulfill this criterion, along with exemplifying the sharing of a whole suite of *Homo* traits with humans.^9^

J. J. Rousseau was one of the first to imply that chimpanzee (in 1755 misnamed “*orangutan*”) had sister species status to humans, inquiring if there was a possibility of accepting them as early “*l’homme*”*,* based on their “human countenance … exact resemblance to man … [with their] potential faculties” – these being language/basic moral reasoning that advanced over time, reaching a climax in humans (for further historic conception of evolution and chimpanzee–human relatedness see supplementary material, sections 1.1., 1.2., 2.1.). Louis Leaky even stated: “Now we must redefine tool, redefine man or accept chimpanzees as human” upon hearing Goodall’s discoveries on chimpanzee tool culture.^10^

## CHIMPANZEE AND BONOBO ECOLOGY, MORPHOLOGY, AND BEHAVIOR

Field studies of chimpanzee (*Pan troglodytes*) field sites began in 1960 by J. Goodall at Gombe and by T. Nishida in Mahale National Park, both sites located in Tanzania. Later sites included Kibale, Uganda; Taï National Park, Ivory Coast; Assirik and Fongoli, Senegal; Bossou, Guinea and Tongo, Democratic Republic of Congo (DRC), and others.^11^ Bonobo (*Pan paniscus*) field research began in the 1970s in Wamba and Lomako, DRC.^12^

*Pan* species (chimpanzees and bonobos) live in rain forests, closed canopy woodlands, open woodlands, and savannahs. *Pan* was divided into two separate species (*Pan troglodytes (P. t.)* and *Pan paniscus (P. p.),* respectively) since 1929, and based on genetics they diverged 1.7–2.7 million years ago (mya). The four chimpanzee subspecies had diverged 1.6 mya: eastern (*P. t. schweinfurthii*), western (*P. t. verus*), central (*P. t. troglodytes*), and central western (*P. t. vellerosus*).^13^ McGrew^11^ referred to them as “races”; S. Rumbaugh^14^ recognized a separate distinct race of “*Koola-kamba*”.^13^ Bonobos display lower genetic diversity than chimpanzees.^13^,^15^ In terms of systematics, until the year 2000, all species of great apes (chimpanzee, bonobo, gorilla, and orangutan) were categorized into the family Pongidae, separated from the human family Hominidae, which was uniquely reserved for humans and extinct ancestors until then (members of *Homo* and Australopithecines). This was redefined with Goodman’s genomic phylogenetic research^6^,^7^ showing that *Pan* is more closely related to humans than to great apes, thus placing *Pan* as the tribe “*panini*” alongside humans as the tribe “*hominini*”, both under the subfamily of Homininae as sister taxa. The other great apes (gorillas and orangutan), with new evidence of genetic affinity to humans, were also included in the family Hominidae^6^ (for further genomic evidence see supplementary material, section 3.).

*Pan* is sexually dimorphic; males are 15% bigger than females as well as in canines and body size.^13^ Bonobos, living only in the Democratic Republic of Congo (DRC), Congo River Basin, differ from chimpanzees in their sharp vocal pitch, agile figure, round crania, and hair parted on the head.^12^,^16^ Chimpanzees live from Senegal and Mali in the west, through the Congo, south to Angola and east to Tanzania, and extending north to Sudan.^12^,^17^ The *Pan* genus is omnivorous, eating fruit, herbs, and hunted meat. In times of shortages, these species will adaptively switch to other foods.^11^ Hunting methods vary with communities and is usually male-initiated. Monkeys are the favored food of chimpanzees, whilst bonobos hunt duiker antelopes.^15^ Hunting may be affected by female receptiveness, food availability, and rivalry.^18^ Furthermore, social organization is ecologically correlated, as seen in savannah chimpanzees in semi-arid habitats, as sociality is more bonobo-like, i.e. having male–female bisexual bonds.^19^,^20^ Community structure in *Pan* consists of a multi-male multi-female structure with subgroupings, ranging from 25 to 150 members. Males stay in their birth group, and females migrate to other communities upon sexual maturity to avoid inbreeding. Group size is affected by ecology, seasonality, predators, competition, and female and food availability.^11^,^21^ At night they all make elevated bed platforms (bonobos cover themselves with leaves).^11^,^12^ Sleeping on the ground^15^ or in caves has been reported.^19^,^20^ Home ranges in chimpanzees vary from 10 km² (Tanzania) to 50 km² (Senegal), depending on population density and resource availability.^11^

In bonobos, inter/intra-community aggressive confrontations are resolved via sexual interactions.^12^,^14^,^15^*Pan* females develop enlarged vaginal swelling when receptive, reach sexual maturity after age 13 with menstruation occurring once a month, and have a 9-month pregnancy. The prolonged period of parent-child dependency enables increased learning and socialization, similar to human patterns.^22^,^23^ Lastly, female bonobos are sexually receptive throughout their cycle (which chimpanzees are not), and bonobo males and females regularly have inter/intra-sex sexual relations (i.e. heterosexual and homosexual relations) (S. Rumbaugh, personal communication, 2010).

### CHIMPANZEE CULTURAL DIVERSITY

The characteristic of human war, according to E. O. Wilson,^24^ matches that of chimpanzees,^25^ suggesting common origin. He defines war as: “Team play, altruism, patriotism, bravery [in] battle, [is] the genetic product of warfare. Consciously ponder[ing] adjacent groups deal[ing] with them in organized fashion, dispose of a neighboring band, appropriate its territory and increase its own genetic representation sense of group identity”. Wilson^24^ also defines “tribalism”: “A tribe should follow a double standard of morality: one kind of behavior for in-group relations, another for out-group”. According to this definition, chimpanzees can be combative tribes participating in warfare, since they follow these criteria. Finally, Wilson^24^ adds: “Chimpanzees … may resemble … ceremonies of earliest man”. As can be seen from chimpanzee cultural diversity, such a suite of traits is not uniquely human but cross-hominin (*Homo/Pan*). “*‘Rites de passage’* cement[s] the ties of the young to the adult group that accepts him”.^24^ Rain dances, waterfall dances,^17^ and fire dances^26^ of chimpanzees can thus be seen as cultural customs, unifying the tribe.

Remarkably, chimpanzee and early human warfare parallels include: adult males patrolling community home-range peripheries, territorial acquisition, and penetrating rival home-ranges. Chimpanzees are known to lynch lone rivals only when victims are out-numbered to reduce patrol injury, with males even holding down their victim, whilst others beat him.^27^ Thus, walking in groups protects against attacks. Taï forest chimpanzees intervene upon hearing cries of their threatened community member, saving him/her physically at great risk to them, or via manipulation by screaming from the forest-cover simulating a counterattack.^25^ A mother with her baby was seen saving the life of her ally when an attempted lynch was being made by another community patrol. Chimpanzees cover their infants to protect them while enduring beatings.^17^,^25^ Intercommunity encounters within Taï forest chimpanzees have been mostly non-fatal, with conflicts resolved by drumming and vocal exchanges. Migrating females with babies visit the ruling male coalition of neighboring communities for protection (culturally unique in Taï).^25^,^27^ Chimpanzee-drumming before a conflict (cultural trait in Taï) is used for communicating across long distances, perhaps as a code differing between communities. Also, Taï females join males on patrols and participate in lynching. Males take nonlocal females captive for up to two days when finding them on patrol, blocking their escape, hitting, comforting, feeding, and sleeping next to them. They release them upon arrival of the victim’s community patrol. Taï females are even known to take other rival females captive.^25^ Similarity with humans is astounding.

Senegal is a semi-arid savannah environment, affecting population density and resource availability of chimpanzee communities. Here, they use water acquisition and purification techniques: e.g. digging wells 50 cm deep in dry river beds or near shallow stagnant pools (in Assirik, Senegal), and, to collect the water, they chew up leaf sponges or make leaf cups.^11^,^28^,^29^ In Bossou, Guinea, chimpanzees have a culturally unique trait of using stone hammers and anvils, stabilizing them via sticks/pebbles, even bringing stones to the communal nut-crushing site.^18^,^30^,^31^,^32^ In Nimba Mountains, Guinea, chimpanzees process Treculia (*Treculia africana*, i.e. “African bread-fruit”) fruits by using stone cleavers and anvils to cut them open. In Gombe (Tanzania) chimpanzees have a “rain dance” custom, which is represented by the members exhaustively running, ripping branches, and swinging repeatedly as the rains begin; in the “waterfall dance”, a chimpanzee leaves the group and gets into a kind of trance beside the waterfall, repeatedly running around it and racing up branches, excited, and even endangering himself (there is no apparent goal in the process, aside from the experience).^17^ In the Mahale (Tanzania) custom, chimpanzees roll over big rocks in a pond becoming excited from the splashes. When young chimpanzees are bored, they make dolls (of small dead animals) and play and carry them for long lengths of time; in Bossou, Guinea, they capture live hyraxes for amusement.^11^

Savannah chimpanzees display cultural traditions affected by adaptation to semi-arid habitats, each culture having distinct community traditions.^28^,^29^,^33^,^34^ In Fongoli, Senegal, J. Pruetz discovered cultural traditions of cave-dwelling savannah chimpanzees who used spears (up to 102 cm long) made from branches with one end sharpened using their teeth or nails to hunt prosimian bush-babies (*Galago senegalensis*).^19^,^20^,^35^ Other reports of savannah chimpanzee (*P. t. verus*) cave-dwelling have been noted from Mali. The caves are used in the hot hours of the day for refuge.^19^ Chimpanzees, limited by resource availability, use calculated ecological knowledge of their habitat to survive. In Ugalla, Tanzania, part of the chimpanzee culture is to unearth nutritional medicinal roots and tubers via multiple digging tools,^36^ demonstrating the capacity for abstract thought and possible mapping of their resources for future reference (the only sign of the tubers above ground are its specific leaves). C. Sanz^37^ and C. Boesch^15^ documented chimpanzees in Goualougo Triangle, Congo, digging honey-bee hives with up to five-part ready-made tool-kits to extract the honey by, e.g., surface breaking, scraping, using an awl and other extracting tools;^38^ even spending half a day smashing beehives with large wooden clubs has been documented. Chimpanzees use shovel tools for ground-breaking (leveraging with their legs),^28^ and stick tools fashioned to fish termites^11^,^17^,^18^,^28^,^39^ (for examples of indigenous peoples’ knowledge on chimpanzee culture and tool-making see supplementary material, section 1.2.).

All these cultures suggest behavioral plasticity of *Pan* to new environments – such quick adaptation is a key trait in *Homo* evolution, particularly after shifting from forest to savannah. Remarkably, chimpanzees may horizontally follow the vertical human shift from forest to savannah. Chimpanzees transfer food/tools across long distances, e.g. water-rich tuber carrying in Tongo, Congo (DRC), and rock carrying in Guinea-Bossau.^15^,^36^ They use “leaf gloves” to squash and eat ants with stingers^40^–^43^ or use long twigs to catch the ants so they can be eaten easily.^17^,^44^–^47^ Moreover, they use leaves as “toilet paper” for cleaning themselves, twigs to clean their teeth, big leaves as a head-cover from rain,^17^,^48^ as well as many other uses. Behavior based on ecological stresses is compiled with ingenuity and exploration to spread culturally distinct behavior, tool production and use patterns across generations.

Lastly, chimpanzees mourn the loss of their dead relatives; an individual may even lose the will to live and may even fall into a depression and die next to their loved one. When humiliated, punished, or attacked, an individual steps aside from the group to cry and be self-reflective^17^ (J. Garen, personal communication, 2009). Chimpanzee cultures are diverse. However, out of 120,000 chimpanzees left, only 49 field sites were studied, leaving much to be discovered.^11^ Chimpanzee parallelism to human behavior is impressive.

### BONOBO CULTURAL DIVERSITY

Little is known about the bonobo’s cultural diversity in nature compared to the highly documented chimpanzee cultures. Long-term cross-population studies need to be conducted in varying ecologies to uncover more information (Nevo, Goodman, personal communication, 2009). Previously, it was thought that bonobos lived in swamp rain forests, but data from Lukuru, the Congo (DRC), field site of J. Thompson, show that they live in savannah habitats as well.^15^ S. Rumbaugh has described bonobos from Wamba (DRC), who leave symbolic markers on trails that navigate lost community members in the direction that the group is traveling (these include broken branches and leaves). Following footprints in the mud is another technique the bonobos use, suggesting recognition of foot shapes that belong to the bonobos^49^ (i.e. bonobo symbolic competency^9^). Food preparation has been seen by S. Rumbaugh (personal communication, 2009) in Wamba bonobos, picking bolingo fruit from trees and letting them ripen for two weeks on the ground before returning to eat them, suggesting a form of primitive harvesting. Bonobos can bipedally walk relatively long distances (around 100 feet), holding food items in both hands with mothers carrying their babies on their chest,^15^ imitating early human behavior.

## SUITE OF BEHAVIORAL *HOMO* TRAITS IN *PAN*

### CHIMPANZEE–HUMAN PARALLEL BEHAVIOR

Behavioral parallelism (e.g. sociality, aggression, and sanctioning) abound between chimpanzees and humans:

#### Political affiliations

Change is based on hierarchical power in chimpanzees (as in humans), with coercions and reciprocity resulting in new alliances to topple leading coalition members. The reward is the access to females and food sites.^15^,^17^ Lower-ranking males reproduce through manipulation, avoiding the coalition, quietly gesturing and branch shaking to lure a female.^17^ Sharing meat within the hunting coalition and females strengthens the community’s ties (hunting roles include: scare, ambush, run after, or capture prey – each member having a defined role).^11^,^15^,^50^ Affiliations of community members are the keys to survival.

#### Social rules

(These differ across *Pan* cultures) In one example, the top-ranking male will not submit via pant grunting to anyone; he usually will not even tolerate eye contact from lower-ranking individuals outside his coalition and not accept being groomed by them either.^51^ According to S. Rumbaugh (personal communication, 2010), all gestures are potentially cultural.

#### Rite of passage

Teenage males are known to transfer from youth groups to adult groups by proving their superiority over the community females, demanding submission from each of the females, even through force, until they bow to him and grunt.^11^ Alliances are constantly rebuilt with grooming, kissing, hugging, and spending time together.^17^ Membership includes protection and sleeping together (holding another’s branch to help him climb or giving a hand to come down has been observed in captivity).^51^

*Aggression* includes biting, beating, dragging, manipulating, screaming, chasing, throwing rocks, carrying/breaking big branches and foliage, hitting with sticks, pounding the ground or tree stumps with hands or feet, and jumping furiously.^17^,^52^,^53^ The context defines the response, e.g., small slaps of a mother to a child who is bothering her or two friends kicking and punching each other to compare strengths.

#### Sanctioning

The taking of food out of turn can result in slaps from other community members^11^ and therefore elicits crying and feelings of shame from the accused.^17^

As in humans, male chimpanzees use lethal violence as a strategy towards neighboring communities to get access to more resources (food, water, and land) and females. Two recorded communities have been eliminated by this: one is the strategic killing of the Kahama community by their neighboring Kasakela in Gombe; the other community is in Mahale.^25^,^52^ They leave their enemies’ dead bodies, arms and legs spread out, missing fingers and testicles as a sign to the victims’ community.^27^,^25^ Castration is a symbolic means of removing male status and lineage in *Pan* (Freud^54^ termed this: “great fear of castration”) and is seen in non-lethal aggression, too.^25^ The high-level cognition of hominins (*Homo* and *Pan*) is used for their benefit. Wrangham^27^ compared chimpanzees to delinquent youth gangs. Both groups target lone individuals of the other communities, retreating when the odds are against them. Intra-community aggression is mostly limited to leadership conflicts between rival subgroups,^15^,^25^ shortening their time in power.^17^ According to Kano,^12^ bonobos have missing fingers, a sign for intra/intercommunity aggression and theoretically a form of punishment (more cases with males compared to females and also rises with age).

### CHIMPANZEE–HUMAN COMPARATIVE EMOTIONAL ANALYSIS

A recent study by Burrows^55^ on human–chimpanzee facial expression comparisons concludes that “In *P. troglodytes* … there is no foundation for claiming greater complexity in *Homo* facial expression musculature. In addition, there are minimal anatomical differences between chimpanzees and humans, contrary to conclusions from previous studies.” All emotions are meaningful representations of socially understood behaviors.^14^,^16^,^49^,^56^–^58^ Facial expressions and gestures – along with eye direction – are contextual means for emotional and informational exchange. Emotions, body language, gestures, facial expressions, and vocalization of intonations seem to parallel between sister species *Homo* and *Pan*. Both S. Rumbaugh^16^ and Fouts^59^ stated how easy it is to understand *Pan* vocal intonations expressing their attitudes, feelings, and body language – in human cultural terms. Human–chimpanzee facial expression comparisons have been conducted using the “chimpFACS” program for analysis.^60^

Examples of *Pan* facial expressions, on par with those of humans, can be seen in an array of shared emotions and behaviors.^9^,^14^–^17^,^59^,^61^

**Figure 2:**
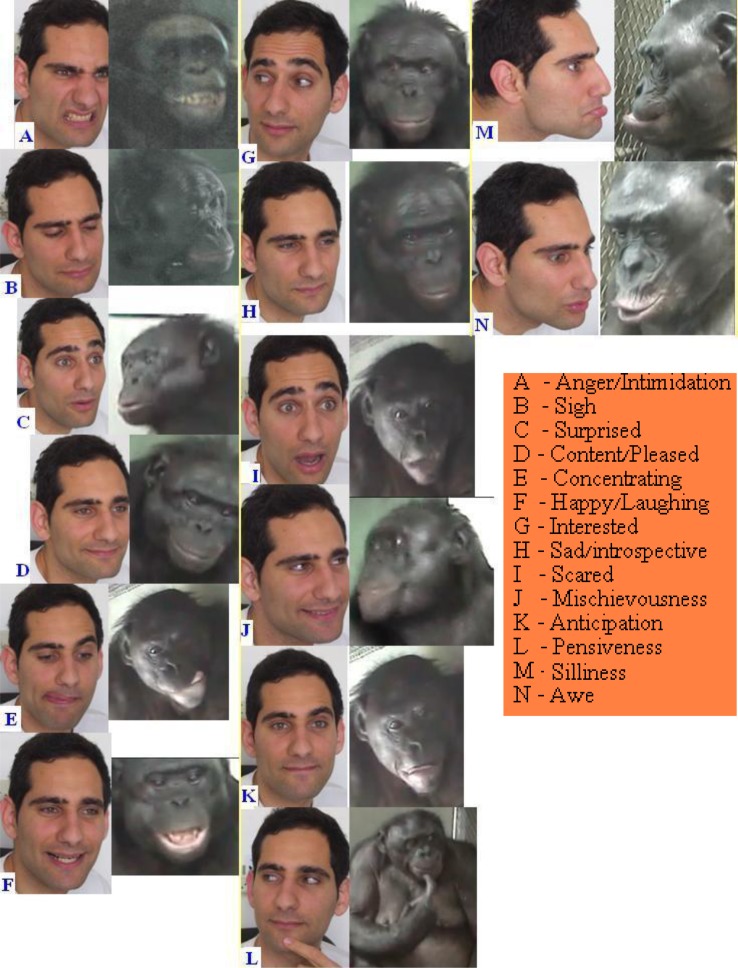
Illustrations of *Homo* facial expressions in *Pan* (Roffman compared with Kanzi and Pan-Banisha)

*Homo* facial expressions in *Pan* include (Roffman, personal observations; adapted with data from Peleg et al.^61^) (Figure 2):

- *Jealousy:* arms crossed over chest, eyes pointing to the side, forehead wrinkled downwards, and lips shrunken.
- *Anger:* eyes peering in intent, eyebrows pointing downwards to the nose, breathing heavily. Hitting the wall, punching, kicking, being uncooperative, and screaming in bass voice (Figure 2A).
- *Crying:* tears, nose running, eyes closed, weeping, face wrinkled, wide mouth closed.
- *Sadness:* chin wrinkled, lower lip protruding (Figure 2H).
- *Shame:* shoulders down, eyes down, body curled in pensiveness (Figure 2H).
- *Happiness:* smiling with mouth wide open, showing just the tip of the teeth, eyebrows up, eyes wide open, usually done along with excitement (hand shaking in the air, jumping in place, or running around screaming high notes) (Figure 2F).
- *Awe:* open mouth, eyes wide open (Figure 2N).
- *Fear:* open mouth showing teeth locked and eyebrows down (Figure 2I).
- *Concentration:* tongue sticking out during fine-motor activity (Figure 2E) (drawing,^9^ tool-making).
- *Mischievousness/manipulation:* eyes directed upwards to the right or left with small grin (Figure 2J).
- *Excitement/anticipation:* standing up bipedally, shifting body weight from left to right, arms waving backwards and forwards, smiling widely (Figure 2K).
- *Grief:* sitting alone, hands on head with eyes closed.
- *Anxiousness:* rocking, moving impatiently and scratching, eyes up, blowing air upwards (Figure 2K).
- *Comforting/love:* holding hands, touching, kissing, hugging, extensive eye contact (Figure 2D).

*Homo* body language expressed in *Pan* (Roffman, personal observation) includes the following repertoire of gestures^16^,^17^ (see Figure 2; see also supplementary material, Figure 3):

- *Go away:* expressed by raising the arm and throwing the hand forward quickly.
- *Come near:* hand waved with the arm in an inwards motion towards their body.
- *Hurry:* shaking both hands fast above their chest (in Kanzi’s case: giving one big clap and pointing to the interpreter to move).
- *Vocal intonations:* laughter – silent quick breaths, vocal grunting, and gargling. In fear the vocal intonations are high-note shrieks. Chimpanzees show surprise by saying “*Waa*”, as in English “*Wow*”; they show frustration by rolling their eyes, blowing air out making bubble noises with their lips. When they are adamant for something to get done they say: “*Wooo*” or “*Oooo*” like in the Hebrew word “וּנ” (“*Nu*” means: *come on*); when making a mistake they hit themselves or the floor (Figure 2N).
- *Gaining one’s attention:* tapping on the back, arm, or knee. *Directing someone* to an event or object by deliberately holding the individual by the hand, pulling them toward or away based on the context (see supplementary material, Figure 3).
- *Disinterest:* expressed by head up, eyes closed, and nose up.
- *Threatening:* eyes directed at opponent, hand hitting the ground, body jerking forward slightly.
- *Disagreeing:* pulling up shoulders.
- *Agreeing:* shaking hand forward and shaking the head vertically.

According to Hopkins,^62^–^64^ chimpanzees have lateralization and handedness like humans, even producing novel vocalizations: “Communicat[ion] … in chimpanzees suggest … neurological substrates underlying language production in the human brain may have been present in the common ancestor of [both]”.^65^

The combined suite of *Pan/Homo* traits uniquely characterizes them and is different than those of animals. By interpreting *Pan* traits in *Homo* behavioral terms, we see they are understandable.^16^ As Darwin said:^66^ “[In] Expression judging I see only one way of testing [it], observe whether the same principle by which expression can, as it appears, be explained, is applicable in other applied cases with satisfactory results”. Emotions, body language, and facial expressions convey information exchange; the slightest of gestures, smirk, eye movements (Figure 2), or scratching has meaning (see supplementary material, Figures 2,3). Chimpanzees, as with humans, read body language to decide whom to trust or avoid. Chimpanzees make friends selectively, a clear survival strategy and expression of high comprehension. Those who have lived with chimpanzees know how suspicious and interpretive they are, and that they will not communicate with those they distrust or fear, as is the case with humans.^14^,^16^,^59^ Wanting to be treated respectfully dictates whether a chimpanzee or human will continue the conversation (in mime or in language, if they are language-competent). Once friendship is established, years can go by and the next time they meet they will express great surprise and joy.^59^

Chimpanzee research in captivity has shown a significant suite of traits compatible with human psychology.^67^,^68^ Post-traumatic stress disorder (PTSD) of varying severity has been diagnosed in sanctuaries for biomedical laboratory-retired chimpanzees. Human psychological pathologies displaying symptomatic behaviors of humans in isolation seen in *Pan* are: 1) obsessive compulsive disorders (OCD), 2) self-mutilation, 3) obsessive hair-pulling (trichotillomania), 4) crouched body rocking, 5) repetitive head banging, 6) spinning around until loss of consciousness, 7) repetitive mumbling and mouth movements, 8) severe depression, 9) dementia or psychosis that may lead to loss of will to live (socialize, eat, or drink), and 10) aggressive sociopathic behavior.^67^–^69^

### SIGN LANGUAGE IN CHIMPANZEES

Bruno, an American Sign Language (ASL)-competent chimpanzee (from Oklahoma ASL cross-fostering program), tragically transferred to biomedical research, was documented from his small cage signing: “*Key, Cut*”. Booee, another language-competent chimpanzee signed: “*Booee, Booee, Me, Booee; Give Me Food Rodge*” (Booee, reunited with R. Fouts, who was his sign-language tutor after 13 years in biomed, remembered his nickname). Further intelligent informational exchange includes Washoe, another ASL language-competent chimpanzee, after hearing that a volunteer had a miscarriage replied: “*cry*, *please, person, hug*”. An ASL-taught cross-fostered female chimpanzee, named Lucy, invented definitions, e.g. watermelon: “*drink-fruit/candy drink*”; radish: “*cry hurt food*”; orange: “*smell fruit*”; celery: “*food pipe*”; sweet pickles: “*pipe candy*”.^59^ Lucy, unlike language-competent chimpanzees and bonobos, did not use her sign language to talk; she simply answered questions about the name of things when asked (S. Rumbaugh, personal communication, 2010). This suggests metaphor and associative thinking. *Pan* exhibit imagination in playing games such as make-believe eating from an invisible plate and spoon, playing with invisible dolls, or inventing imagined enemies to attack^14^,^16^,^59^ (Patterson at www.koko.org has shown informational exchange by an ASL-competent gorilla, Michael, who gave testimony about how his mother was killed in Africa).

### *PAN* VERSUS *AUSTRALOPITHECUS* MORPHOLOGY

E. Mayr^70^ states: “Australopithecines … intermediate between chimpanzees and *Homo* … [having] the total assemblage of their characteristics … closer to chimpanzees ... [in] arboreal[ity] … sexual dimorphism [and] brain [size of] 450 cubic centimeters” (cc). Bonobos have body proportions like Australopithecines (early *Homo*), differing only by the degree of adaptation to bipedalism.^12^ Bonobos and *Australopithecus afarensis* (“Lucy”) have identical cranial capacity and skull.^71^ Bonobos are more slender than chimpanzees, have a tendency for bipedalism, longer legs, longer necks, less body hair, stronger leg muscles, alternating division of body mass, elongated foot bones, and a spine that enters the skull lower than in chimpanzees, all of which are Australopithecus-like traits^12^ (see supplementary material, Figures 2–5, 7).

Our hominin family tree (see Figure 1) urges us to redefine *Homo* to include our closet relatives. Species demonstrating the suite of traits of another should be accepted to join this genus. This criterion suggests that *Australopithecus* be placed into a subgenus under one genus: *Homo. Homo habilis* is more comparable to *Australopithecus* than to *Homo*.^6^,^12^,^72^,^73^*Australopithecus afarensis*, “Lucy”, had intermediate characteristics of *Homo* and *Pan*.^74^ Lucy’s *Pan*-like tree climbing tendencies can be conferred from her elongated curved feet and hand finger bones (phalanges), as seen in *H. habilis* as well. *H. habilis* kept multiple physical features present in Australopithecines: short ∼1 meter height is another *Pan* trait along with a *Pan*-sized brain in *A. afarensis* (438 cc) and *H. habilis* (363–580 cc).^75^–^77^ Australopithecines share a suite of traits with *Pan paniscus,* especially in anatomy.^72^,^78^ According to muscle morphology and bone density, Australopithecine strength was the same as in *Pan* (seven times stronger than humans).^73^ Could *Pan* be a living fossil of Australopithecines? (For elaboration on hominin phylogeny and speciation of *Homo* genus see supplementary material, sections 2.2., 2.2.1., and Figure 1 and Table 1 of the supplements.)

**Figure 3:**
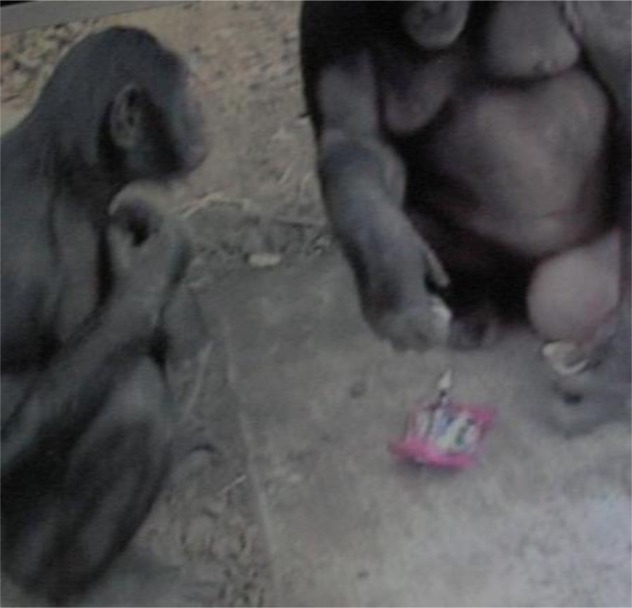
Bonobo Pan-Banisha (left) holding a flint flake

**Figure 4:**
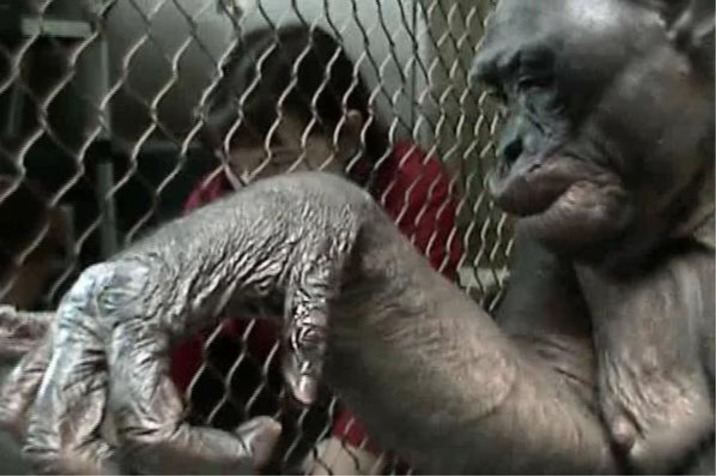
Bonobo Pan-Banisha’s hand exhibits fine motor ability and manual dexterity.

The following gives further examples supporting *Pan paniscus* as a living Australopithecine: *Australopithecus afarensis* has a cone-shaped thorax like *Pan*, with shoulder and back muscles involved in arboreality.^79^,^80^ These morphological changes are shared between *Pan* and *Australopithecus*. Australopithecines express traits of *Homo* in hand structure (in connecting thumb and fingers for delicate motor skills needed for stone tool-making). Kanzi and Pan-Banisha exhibit such competency in basic Olduwan flake production (see Figures 3,4).^9^,^81^ Hence, Australopithecines should be accepted as variations within the *Homo* genus with *Pan* as a sister species and subgenus under *Homo. Pan paniscus,* based on shared traits with *Australopithecus*, needs to be included in Australopithecine’s subgenus. Australopithecine’s intermediate relation between *Homo* and *Pan* should support bonobos as Australopithecine, just as *H. habilis* is *Homo* (for elaboration on *Homo* genus speciation, see supplementary material, section 2.2.1., Table 1; including the newly discovered fossil of *Ardipithecus [Australopithecus] ramidus* suggested to be a *Pan–Homo* missing link that is very bonobo-like).

Regarding adaptation to a savannah ecotype, bonobos in Lukuru, DRC, who are very vocal in the forest, are silent in an open savanna, implicating early *Homo* behavior. Thompson in Lukuru has noted that bonobos, at the first sign of intruders, duck down from their bipedal walking in the open savannah, escaping via knuckle-walking.^15^ Foraging and sleeping in the grasslands at Lukuru suggests bipedalism is adapted with access to different ecotypes for acquiring new resources.

## COMMUNICATION BETWEEN *PAN* AND *HOMO*

The *Pan/Homo* culture at the Great Ape Trust of Iowa (GATI) provides a sanctuary for bonobos to express their full potential. Kanzi (male, age 27; see supplementary material, Figure 2) and Pan-Banisha (female, age 26; see supplementary material, Figures 3,4,5), both English lexigram language-competent bonobos, convey their thoughts via a 450-symbol computer keyboard by combining short sentences and understand 2,500 English words. Activities at the sanctuary include: exploring the forest, harvesting, making complex tools, sleeping outdoors, expressing their culture whilst communicating in human language^81^ (see also supplementary material, section 4, Figure 6).

### INFORMATIONAL EXCHANGE WITH LANGUAGE-COMPETENT BONOBOS: KANZI AND PAN-BANISHA – PERSONAL EXPERIENCE

Like humans, individual chimpanzees/bonobos have personal identity, hobbies, interests, unique personality,^82^ lifelong memories, creativity, and a high mental competency potential that have only recently been discovered.^9^,^16^ Bonobos/chimpanzees who are allowed to live in a language-rich environment develop language and symbolic competency.^14^,^81^ The lexigram English language-competent bonobo, Pan-Banisha, showed her capacity to see from another’s perspective (bifurcated consciousness), when she empathically observed a new worker at the Language Research Center, Atlanta, Georgia, USA, who was blind; Pan-Banisha, in contemplation, continuously walked around with her hand covering her eyes to feel what it was like to be blind (personal communication with S. Rumbaugh, 2008). “Selfhood” is having the capacity to comprehend how others see things, different from one’s self and relating to the others.^68^,^83^

I. Roffman has spent two years with language-competent bonobos, Kanzi and Pan-Banisha, for his Masters thesis research in anthropology^9^ on *Pan/Homo* intelligent informational exchange at the Great Ape Trust of Iowa. S. Rumbaugh^14^,^81^ established this *Pan/Homo* culture of a bonobo family living with a human family in a language and culture-rich environment in the 1970s. Kanzi and Pan-Banisha acquired language untrained, simply from growing up in an experiential communication setting in the forest. For his research, I.R. spent days on end with Kanzi, watching his favorite movies, playing, drawing, and sharing food while conversing with one another via computer with a 450-word lexigram keyboard. On one memorable occasion, Kanzi asked I.R. to make a meal for him with particular ingredients for a salad: “*lemon juice, lettuce, sugar, salt, raisins, onions, and celery*”. When I.R. forgot to add the raisins in the bowl, Kanzi repeatedly said “*raisins*”! Kanzi also requested I.R. to dance with him. He would gesture to do it again by making a circle motion with his index finger, even pointing to which direction to turn, sit down, or get up. These were bilateral conversations, contextually appropriate. Kanzi would rhythmically drum on his ball as I.R. would sing. I.R. once played an audio clip of Ofra Haza (Israeli folk singer; song “*Yad-Anuga*”/“*Delicate Hand*” in Hebrew) in the presence of Nyota (see Figure 7 in the supplementary material), Pan-Banisha’s 12-year-old son, who joined in tune with his high-pitch vocals in the right places in the song, and as the melody rose in impact, so did his vocals. He used a bowl as a musical instrument by sliding it on the floor as the song played, making rhythmically appropriate swooshing sounds.

Kanzi and Pan-Banisha (Figure 3) have demonstrated a sense of humor on many occasions; to name a few: 1) Pan-Banisha called I.R. “*Quiet Gorilla!!*” whilst pointing to him, referring to his talking (the term “gorilla” is known to Pan-Banisha as a monster, to warn her from going to dangerous places in the forest^16^). 2) Kanzi (see supplementary material, Figure 2) laughed when I.R. was requested by him to dance in place and turn around until he was exhausted. 3) Pan-Banisha (see supplementary material, Figures 3, 4, 5) asked I.R. to hide in a game she initiated of “hide-and-seek”, knowing he was under the table, upon his request to return, she laughed and disagreed, forcing him to wait there for 15 minutes. 4) On the first few days of meeting the Wamba bonobo family, Kanzi’s half-brother Maisha (age 7) requested I.R.’s small notebook. Upon giving it to him, Maisha made a manipulative facial expression, his eyes looking to the right with a smiling grin. He then acted as if he was going to return the notebook by putting it on the floor. However, the moment I.R. touched it, Maisha left the room and went into Kanzi’s room to tell him about what happened, and Kanzi came out with a cup full of water pouring it on I.R.

### TESTIMONIES OF KANZI AND PAN-BANISHA LIVING IN SANCTUARY

Both Kanzi and Pan-Banisha reflect on past events that impacted them personally, and convey informational narratives, sharing testimony with their human friends and family. Their reports on events are contextually accurate; they combine words logically to convey past events in present and future references:

Pan-Banisha shared her testimony on the passing and cause of death of bonobo P-suke, father of her children. She stated in apparent anguish: “*P-suke, P-suke, Shot, Electric Shock*”. When I.R. asked what she was talking about, a chronology of events unfolded, which was described in her short testimony. “*P-suke*” suffered from a hernia and was sedated with a needle (“*shot”*), but he had a bad heart condition and his heart stopped, thus necessitating an electrical shock on his chest (“*electric shock*”), which sadly caused his death. Pan-Banisha not only expressed her great grief at remembering the loss of her family member by crying and screaming, but shared this information with I.R., who did not know what had happened – giving a valid testimony (I.R. observation, 2007) (The Great Ape Trust of Iowa staff and workers did all they possibly could to help save P-suke). The capacity to share complex abstract chronological information, reflecting events from the past, was thought to be a uniquely human intellectual ability.

Kanzi conveyed similar competency in sharing his testimony that was unknown to the listener (I.R.) prior to hearing it. Here, Kanzi said: “*Hose, Hurt, Kanzi*”. I.R. asked Kanzi’s guardian, S. Rumbaugh, if the testimony was valid, which she said it indeed was. Kanzi had the occurrence happen several years before, at night, when the staff at the “Language Research Center” was not there, when he was hurt on his hand by a hose.

## CONCLUSIONS

We overviewed the relationships of humans and chimpanzees historically and showed that the Darwinian view-point is the only sound view relating man to mammals, primates, and great apes. Darwin extensively documented phenotypical information, and later research substantiated, both genotypically and phenotypically, that humans evolved gradually from apes, sharing a common origin with chimpanzees and current great apes. Extensive field research (since the mid-twentieth century), novel fossil evidence, and remarkable genomic studies suggest that chimpanzees and humans are hominin sister taxa (see supplementary material, sections 2.2., 2.2.1., and 3.). The seemingly remaining gap between humans and chimpanzees, i.e. language competence and symbolism, is also bridgeable and suggests that even on this last issue the dogmatic view that man is linguistically and symbolically unique is unjustifiable. Hence, even the last fortress of man’s uniqueness is subject to Darwinian gradualism. The data reviewed here support a sequential and gradual connection between the cultural world of the chimpanzee and that of humans. The differences between chimpanzees and humans, despite their genomic great similarities, are explainable genetically but still await further clarification. Some of them are discussed in our future research prospects section (see supplementary material, sections 4, 4.1.).

## Supplementary Material

Supplementary Material

## Abbreviations:

ASL: American sign language
DRC: Democratic Republic of Congo
mya: million years ago
PTSD: post-traumatic stress disorder
