# Supplementary material for: Can Chimpanzee Biology Highlight Human Origin and Evolution?
Source: Rambam Maimonides Med J. 2010 Jul 2;1(1):e0009. doi: 10.5041/RMMJ.10009 (PMC3721662; doi:10.5041/RMMJ.10009)

Supplementary Material

This appendix has been provided by the authors to give readers additional background reading and information about their work.

Supplement to:

Roffman I, Nevo E. Can Chimpanzee Biology Highlight Human Origin and Evolution? RMMJ 2010;1 (1) :e0009. DOI:10.5041\RMMJ.10009

SUPPLEMENTARY APPENDIX – TABLE OF CONTENTS

1. Introduction: the big Darwinian overview on origins and evolution
   1.1. The descent of man
   1.2. Indigenous and hunter-gatherer views on man's place in nature
2. The evolution of evolutionary theory
   2.1. Key chronology of man's conception
   2.2. Discovering the prehistoric human family tree: Darwin's hypothesis realized
3. Genetic evidence relating chimpanzees, bonobos, and humans
   3.1. Human-unique genes
4. Future research
   4.1. Prospects
5. References
6. Table 1. Australopithecine and *Homo* fossil discoveries
7. Figure 1. Hominin (*Pan, Homo,* and *Australopithecus*) phylogeny
8. Figure 2. The bonobo Kanzi (*Pan paniscus*) profile
9. Figure 3. Bonobo Pan-Banisha explaining herself via manual gesture (note joint index finger and thumb along with eye contact) to her guardian, Professor S. Rumbaugh
10. Figure 4. Pan-Banisha exhibiting *Australopithecus*/early *Homo* features (head and body proportions)
11. Figure 5. Bonobo Pan-Banisha exhibiting early *Homo*/Australopithecine features.
12. Figure 6. Envisioned illustration of lifetime chimpanzee sanctuary on Mount Carmel, Israel
13. Figure 7. Nyota (note the *Homo*-like body features)

1. INTRODUCTION: THE BIG DARWINIAN OVERVIEW ON ORIGINS AND EVOLUTION

Evolution is the most fundamental idea man has generated concerning the interlinked cosmos, life, and culture. Evolution highlights our origin and development in space and over time. It relates matter, life, and conscience. Evolution provides insight into the real world, which is ever-changing, including humans. It is also the only rationale for planning the future of life and man. Evolution is the core of all sciences – physical, biological, and humanistic. While physics and chemistry answer the question "how", evolutionary biology answers the question "why". The core of evolution is the ever-continued adaptation to stressful and changing environments. Nevertheless, natural selection is the key evolutionary driving force in the differentiation of species that adapt better to their environments, bringing about speciation either allopatrically or sympatrically. Moreover, the natural evolutionary process is dramatically imitated by the human process of domestication by artificial selection.

Evolutionary biology tells us that life has autonomously diversified on earth without any kind of external guidance. Darwinian evolution has no ultimate purpose or goal. The exclusive raw materials of evolution are variation and natural selection generating adaptive complexes, complemented by genetic drift and gene flow. Humanity originated essentially from the dust of stars. Like all other organisms, we humans descended from prokaryotes and animals by the very same blind forces that created all other organisms; thus we remain a member species of the planet's biodiversity. From a geocentric world, Copernicus led us to a heliocentric world, and cosmology led us to the galactocentric world-view. But the big shattering of the anthropocentric world-view peaked in the Darwinian view of the origin of man. Darwin shed light on the extraordinary evolutionary process by saying: "There is grandeur in this view of life, with its several powers, having been originally breathed into a few forms or into one; and that, whilst this planet has gone cycling on according to the fixed law of gravity, from so simple a beginning endless forms most beautiful and most wonderful have been, and are being, evolved".[1]

1.1. THE DESCENT OF MAN

As concluded by Darwin, man descended from a less highly organized form. Evolutionarily, man displays individual variation and is subjected to on-going selection, as is the case for all organisms (see Templeton in this issue). His phenotype is determined by his genotype interacting with the environment. Natural and sexual selection shaped man's evolution. DNA, RNA, and proteins drive the ontogeny and phylogeny of man, as they drive the development of other organisms interacting with environmental variation and stress, which is primarily climatic.

In fact, the entire evolution of man is determined by the climatic factors of savannization. Man diversified geographically into climatic races from the African common stock, which is the very area where chimpanzees and humans diverged from a common ancestor several millions of years ago. Are the high intellectual and moral standards of man unique, or do they have their origins in his animal heritage? Can intellectual potential and moral standards evolve? Did language, tool-making, and culture generally evolve as adaptive complexes for survivorship? Did the higher faculties of man such as mind, rationality, abstraction, self-consciousness, imagination, curiosity, wonder, past memory and future planning, foresight, creativity, innovations, and feelings of pain, happiness, and misery evolve by natural selection along with his brain to improve social survival? It was the unique traits of society, culture, territorial exclusion and acquisition, ingenuity, invention, tool development, reflective memory, hunting strategies, reasoning, associative thinking, co-operation, wearing disguises, protection from the elements or predators, food acquisition and preparation practices that benefited man's survival.[2] Control of fire, language, and knowing the different species of plants and animals to keep away from or to use for their benefit are paramount in human evolution.[3]

In his *Descent of Man* Darwin[3] showed that there is no fundamental difference of any kind between man and higher mammals in their mental faculties. As Darwin and other observers noticed, man and higher animals, especially great apes, "All have the same senses, intuitions, and sensations – similar passions, affections, and emotions, even the more complex ones; they feel wonder and curiosity, they possess the same faculties of imitation, attention, memory, imagination and reason, though in very different degrees".[3] Darwin's theory was made before fossilized members of *Homo* were found – showing his genius by hypothesizing man's African origins: "Man … descended from the hairy and quadruped … arboreal in habits",[3] and evolved to the current human race by multiple waves of migration from Africa to other continents.[4-7]

1.2. INDIGENOUS AND HUNTER-GATHERER VIEWS ON MAN'S PLACE IN NATURE

The *Pan–Homo* (chimpanzee–human) continuum was well known to human indigenous tribes living sympatrically with chimpanzees via folklore. The chimpanzee, as human 'sister species' in evolutionary taxonomy,[8] is an accepted term by Central African tribes. "*Chimpanzee*" in the language of the

pygmies means "pseudo-human"[9] and being closely related to man and not animals.[10] The Mende tribe of Sierra Leone highlighted chimpanzee culture, which had not been known to science until the ground-breaking observations of Jane Goodall in the early 1960s at Gombe, Tanzania. Observations of the Mende were documented by westerners in 1920; they spoke of tool production and use, manipulation, and politically power-driven chimpanzees who medicated themselves via botanical knowledge. This was validated and expanded by Goodall and other researchers on chimpanzee culture.[11-29] Thus, chimpanzee cultural diversity became accepted,[13] although African tribes acknowledged it earlier in the millennia. In Mende tribal thinking, chimpanzees shared traits with humans in social behavior and cultural structure.[10] This tribe feared and respected the chimpanzees because they were portrayed as the darker side of man by their preconceived motives to harm and kill their enemies.[10, 30]

Regarding tool production, the Mende chimpanzees used tools for social reasons, not just for specific tasks. They described social chimpanzee food processing sites with division of labor, where youngsters learned social rules and obedience, with grown-ups collecting nuts whilst others opened them with stones.[10] Matsuzawa[19,20] documented this in Bossou, Guinea, showing anvils and hammers being used by chimpanzees as multilevel tool-kits.[12,14,19,30,31]

Chimpanzee pre-planned social killing, hunting, and fishing were known to the Mende who witnessed chimpanzees creating mud barriers with a stick to grab fish[10] (S. Rumbaugh, personal communication, 2010: Wamba pygmy tribes in the Democratic Republic of Congo (DRC) describe bonobo fishing practices). Indeed, adult chimpanzees on patrol killing and mutilating non-resident individuals have been recently reported.[14,32] Chimpanzees, like humans, define enemies that are socially acceptable to kill (i.e. non-members of their own community). "We" and "they" is a distinct concept in chimpanzees, as in humans, and must have evolved in our common ancestors. The chimpanzees' care and love for their own community members is seen in their medication practices, such as placing either chewed or squashed leaves on an area of their infant's body infected by parasites.[10] Grown-ups attempt to cure even internal illnesses in youngsters. Huffman[23] documented plant medication, such as folding or rolling specific leaves in a particular fashion, depending on the type of cure for stomach parasites. Adult chimpanzees were seen rolling a spiny leaf into a cigar shape with the smooth surface on the outside and spines pointing inward, deliberately shoving it into the throat of the sick to cure diarrhea or parasites. This documents ecological knowledge and cultural teaching in chimpanzees.[33]

The Mende traditionally call chimpanzees "*Numu Gbahamisia*" or "the other persons", associating chimpanzees with themselves, both originating from "those who walk on two legs". Other tribes living sympatrically with chimpanzees also have traditional names expressing their sister relation to humans (personhood), e.g. in the tribes of the Ivory Coast: the Baoulé call them "the brother we love"; the Oubi tribe calls chimpanzees "unsightly persons"; the Bété tribe know them as "wild forest men"; and the Bakwé give them human burial rites.[10] For physical comparisons of bonobo-chimpanzees with early humans see figures 2, 3, 4, 5, 7.

2. THE EVOLUTION OF EVOLUTIONARY THEORY

2.1. KEY CHRONOLOGY OF MAN'S CONCEPTION

In 1261, Albert the Great categorized animals and man in progression with souls of gradual perfection: the top being rational man and apes below him, as "*similitudines hominis*". Nicholas Tulp was the first in Europe to analyze a chimpanzee in 1641, noting shared traits between ape and man.[10] Also, in 1641, Rene Descartes separated man from animal and nature by divine descent with man having a mind: "I think therefore I am",[27] with all animals described as "mechanical". Hence, he saw no ape–man continuum. As long as religion reigned over scholarship, the singularity of man was held superior. Edward Tyson made the first anatomical dissection of the chimpanzee in 1698. Based on brain composition, he pondered whether chimpanzees could also talk. In 1735 Carl Linnaeus wrote *Systema Naturae* showing the close association of man and great apes by levels of grades, inventing the classification of "*Primates*" and "*Homo*" genus. In the mid-1700s, Georges Buffon rejected Linnaeus' placement of human into the category of primates, as he felt man was created for a reason. In 1779, anatomist Johann Friedrich Blumenbach designated the species "*Homo sapiens*", which differentiated man from great apes based on bipedalism, setting man in distinct order of "*Bimanus*". In 1820 the anatomist Wilhelm Vrolik associated man with great apes in brain structure and in mental progression.[10,34]

2.2. DISCOVERING THE PREHISTORIC HUMAN FAMILY TREE: DARWIN'S HYPOTHESIS REALIZED

Darwin predicted fossil hominids but never saw them. In Darwin's time the only *Homo* genus fossils known were those of the Neanderthals. It was only after Darwin's passing that Eugene Dubois

found *Pithecanthropus erectus* (*Homo erectus*) in Java (1891), making it the earliest *Homo* member to be discovered at the time. Later, in South Africa, Raymond Dart[35] discovered "*Taung child*" in 1924, proving African hominid origins of man with this fossil, described as "ape-man" *Australopithecus africanus*.[2,34,36] In 1938 Robert Broome found *Australopithecus (Paranthropus) robustus* in South Africa. Through stratigraphic faunal association, a mosaic environment of open woodland and riverside grasslands was described for *Homo* origins as Darwin predicted. In 1959 Louis and Mary Leaky uncovered *A. robustus boisei,* and in 1964 they found *Homo habilis* at Olduwai Gorge, Tanzania.[34]

2.2.1. FOSSIL EVIDENCE: SUBSTANTIATING DARWIN'S PREDICTIONS

Dramatic early human fossil discoveries have been unearthed primarily during the twentieth century following Darwin's expectation[1,3] (see Table 1 for a full description). The earliest hominid in the fossil record is *Sahelanthropus tchadensis* from Chad, Africa, dated 7-6 million years ago (mya), exhibiting an extreme example of mosaic evolution of primitive character (small brain in a small body) and hominid characteristics (small canines). The hominin ancestral lineage (see Figure 1) includes *Ardipithecus ramidus* (4.4 mya) from Gona, Ethiopia, which is thought to be a direct ancestor to Australopithecines. With 300–350 cc brain volume, facial prognathism, a non-obligate terrestrial biped (*Pan*-like opposable toes), and an arboreal quadruped with decreased canines, *Ardipithecus* was suggested as the earliest hominin.[37,38] *Australopithecus anamensis* was found in Kanapoi, Ethiopia (4.2–3.9 mya). *A. afarensis* ("Lucy") of Afar, Ethiopia (3.2 mya, cranial capacity 438 cc), 105–120 cm in height, was a partial biped with partly opposable toes (between *Homo* and *Pan*). *A. garhi*, of Bouri, Ethiopia (2.5 mya, crania 450 cc), was found contemporaneous with Olduwai tools.[34,36] Subsequent fossils are *A. africanus* of Swartkrans, South Africa (2.5 mya, crania 454–520 cc), exhibiting relatively less sexual dimorphism. *Paranthropus robustus* of Taung and Swartkrans, South Africa (2–1.6 mya, crania ~550 cc), was contemporaneous with primitive bone tools used for tuber excavation.[36] *P. boisei* of Olduwai, Tanzania (2.32–1.41 mya, crania 550 cc), had a relatively flatter face, *Homo*-like positioned foramen magnum, and strong sexual dimorphism (males 1.3 meters tall and females 1.05 meters tall; males 68 kg and females 45 kg).

Homo floresiensis, from Flores, Indonesia (90,000–18,000 years ago), was a biped standing 1.06 meters tall (crania 380 cc) – exhibiting Australopithecine and *Pan* crania size, height, and hand carpal bones.[36,39] As it was found with Olduwai-type tool technology, we suggest that such competencies may be present in *Pan* and Austalopithecines.

The genus *Homo* evolved in east Africa with *Homo habilis* (2 mya, crania 600 cc), along with *H. rudolfensis* (~2 mya, crania 700 cc) from Koobi Fora, Kenya. This species used Olduwan stone tools.[39] An important evolution took place 2 million years ago in *Homo*,[40] from decreased sexual dimorphism, bigger statures, smaller teeth, larger brains, and the transformation of limbs to bipedal specialization.[41] *H. ergaster* (1.8 mya) was 1.7 meters tall, had elongated legs with a foot arch for more efficient bipedality, and with neck muscles and an Achilles tendon for sprinting;[36] their cranial capacity increased from 870 cc (in Nariokotome, Kenya) to 1,000 cc in *H. erectus* (in Asia).[42] *H. ergaster dmanisi* (crania 650 cc) of Georgia is an earlier form of *H. erectus*.[2] *H. heidelbergensis*, (crania 1,200–1,300 cc) arrived in Europe 500,000 years ago from Africa and had diverged into *H. Neanderthalensis* and later in Africa to *H. sapiens sapiens*.[2,34] Europe's extreme climatic shifts caused a cold-climate adaptation 130,000 years ago in the emergence of a cold-adapted *H. Neanderthalensis*.[2,42] Lastly, *H. sapiens sapiens* originated 195,000 years ago in Omo, Ethiopia. The global *Homo* diffusion is a result of climatic shifts allowing long-distance migrations[43] and multiple migrations from Africa.[4,5,7,41]

3. GENETIC EVIDENCE RELATING CHIMPANZEES, BONOBOS, AND HUMANS

Leading chimpanzee/human genome researchers, Goodman, Wildman, and colleagues of Wayne State University, have compiled evidence for including chimpanzees (*Pan*) as a subgenus of *Homo*: "This functional DNA evidence supports two previously offered taxonomic proposals: family *Hominidae* should include all extant apes; and genus *Homo* should include three extant species and two subgenera, *Homo (Homo) sapiens* (humankind), *Homo (Pan) troglodytes* (common chimpanzee), and *Homo (Pan) paniscus* (bonobo chimpanzee). Phylogenetic branching pattern sister groups humans and chimpanzees. The evidence call[s] for grouping chimpanzees and humans together as sister subgenera of the same genus and justify that chimpanzees can provide insights into distinctive features of humankind's own evolutionary origins".[44] Based on molecular-genetic evidence, chimpanzees (*Pan*) are more closely related to *Homo* genus than to gorillas (*Gorilla sp.*).[8,45] The family grouping of chimpanzees and gorillas with humans, based on serum protein immunological intraspecies comparisons, was first proposed by Goodman in 1962.[46-48]

Homo and *Pan* may have separated from a common ancestor as late as 4.8 million years ago, based on late divergence dating, sharing 99% of their genome (99.4% identity in non-synonymous

DNA, and 98.9% identity in synonymous functional DNA). Even the *Homo* and *Pan* cognitive-based brain genes are closer between *Pan–Homo* than to the great apes.[8,49] The chimpanzee–human sister species classification is supported by over 50 years of research in the fields of chimpanzee cultural diversity,[12] language competency,[26,28,50-52] genomics,[8] anatomy, high cognition, psychology, society, self-consciousness and relation to others, tool use/production, *Homo*-level emotions, symbolic competency, memory recollection, complex multifaceted problem-solving capabilities, and interspecies communication.[27,53] These findings also indicate that the cognitive basis of language evolved in *Pan*.[8]

3.1. HUMAN-UNIQUE GENES

FOXP2 is regarded a uniquely human linguistic gene that evolved during the past 250,000 years of human evolution. This gene in humans is thought to control and influence language, as humans who exhibit a mutation in this gene suffer from language disorders and grammatical disabilities.[54] Perhaps earlier *Homo* had such disabilities. This gene differs in humans by only two amino acids from chimpanzees. Other gene sequences thought to be unique to humans include the MYH16 muscle-specific myosis pseudo-gene; HAR1F (human accelerated regions), which develop the neuro-complexity connection networking levels in the brain (implying mental complications in humans including a mutation in it) along with ASPM (Abnormal Spindle-like Microcephaly-associated)
and MCPH1 (Microcephalin).[8,36,55] It is thought that the minute 0.6%–1.1% genomic differences between humans and *Pan* cannot account for the big difference in mental processing and physiological characteristics.[56] These differences could result from regulatory expression via "switch-on/-off proteins" on transcription factors that activate or silence gene expression, affecting such dramatic changes in anatomy and mental development.[55] Additional genetic phenomena might explain the *Homo–Pan* (human–chimpanzee) phenotypic differences.[57] These may include: gene splice variation, generating different proteins, gene transfer, SNPs (single nucleotide polymorphisms) and genome associations by linkage disequilibria, RNA editing,[58] protein intrinsic disorders and cell signaling, and network interaction of proteins.[59] Among important changes in the ancestry of both humans and chimpanzees, but to a greater extent in humans, are the unregulated expression profiles of aerobic energy metabolism genes and neuronal function. These and related brain-related genes, can increase neuronal activity requiring increased energy supply.[49,58]

It is the phenotypic neuro-plasticity and flexible genetic adaptation through expression of the vast potential of gene sequences that unify members of the Homininae subfamily (*Homo/Pan*). We hypothesize that it is the activation, regulation, expression, and organization of genes (99.4% identity in their genomes), triggered by environment and need, that make humans and chimpanzees more similar or different, thus controlling the specific suite of *Homo–Pan* traits. Combining the molecular genetic data by Goodman et al.[8,44] with field observations of the savannah–dry habitat of the bonobos' culture together with captive data on Kanzi and Pan-Banisha's high-level mental competencies, will uncover the neuro-plasticity of *Homo* traits in *Pan*. This may bring these sister taxa species even closer together by suggesting that they differ mainly in gene expression and regulation,[58] thereby uncovering key developments in human evolution.[8,49]

4. FUTURE RESEARCH

The information contained in the main article necessitates the emergence of a new research paradigm towards *Pan* in light of our sister species relationship, taking into account chimpanzee and bonobo cultural heritage and shared psychology with humans. Hence, in captivity, only sanctuary enables them to express their previously underestimated high-level mental capacities, full potential, and communicate their thoughts; allowing them a cultural life of adventurous and experiential activities with the liberty to choose what they want. We propose to establish a lifetime chimpanzee sanctuary (Figure 6) on Mount Carmel, Israel, where there has been continuous *Homo* habitation for more than a million years.[60,61]

4.1 PROSPECTS

The International Graduate Center of Evolution, Israel, will shed light on quantitative cultural and genomic similarities and differences between humans, bonobos, and chimpanzees in a comprehensive research program:
1) Together with W. M. Fields and S. Rumbaugh of the Great Ape Trust of Iowa, USA, and J. Garen of the Wales Ape & Monkey Sanctuary, UK, we intend to document the suite of *Homo* genus traits in *Pan* through voluntary chimpanzee/bonobo communication (informational exchange, giving testimony via English lexigram keyboard, miming, art, music, tool-making, food preparation, high mental competencies, symbolism, selfhood, self-interests, moral reasoning, imagination, creativity, intelligent bilateral communication, complex high-level emotional functioning, language competency, and cross-

cultural exchange).
2) We will incorporate bilateral communicational, informational, and cross-cultural exchange between language-competent bonobos (at Great Ape Trust of Iowa, USA) and young people with severe autism and medium retardation in an inter-hominin (*Pan–Homo*) communication program via novel information technologies and video conferencing software between USA and Israel. We also plan to establish a lifetime sanctuary on Mount Carmel, Israel (see Figure 6), where chimpanzees and bonobos will be able to live in semi-wild African savannah-like sanctuary where they can revive their cultural diversity and have freedom of choice and full experiential enrichment, exposing the chimpanzees' full potentials. Here, a *Pan–Homo* cross-cultural exchange center will be established where they can develop bilateral exchange with communicationally delayed humans, thus advancing voluntary methodologies for delayed children by forming a creative intelligent bilateral interaction for both chimpanzees and children to enrich each other.
3) In addition, the full genomic sequencing of Kanzi is currently being processed by the group of M. Goodman and D. Wildman at Wayne State University in Detroit, Michigan, USA, to assess the genomic divergence of bonobos, chimpanzees, and humans. This genomic study will attempt to explore different bonobo communities in the Congo Basin (Democratic Republic of Congo) to evaluate their cultural and genomic geographic variation, to compare and contrast DNA samples from Wamba bonobos living in swamp rain forests with bonobos living in dry forest savannahs. By doing so, we will analyze genetic differences, implicating speciation, adaptation, and evolutionary changes between separate populations – thus shedding light on human/chimpanzee evolution and genetic adaptational phenotypic neuro-plasticity in the bonobos (*Pan paniscus*). Complementarily, the group of G. Rechavi at Sheba Medical Center, Israel, compares brain gene editing of human and non-human hominins (bonobos and chimpanzees), attempting to unfold by RNA editing methodologies their quantitative cognate potentials.
4) Lastly, in the Congo (DRC) local tribes have known for ages about bonobo/chimpanzee[62] cultural diversity and the suite of *Homo* traits in *Pan*. We wish to include Congolese students and scholars under the International Graduate Center of Evolution. This endeavor could help preserve the endemic chimpanzee/bonobo cultures, which are threatened with extinction, and will expand the scientific understanding of human–chimpanzee sister species evolution.

5. REFERENCES

1. Darwin C. The Origin of Species. London: Dent; 1859.
2. Wolpoff MH. Paleoanthropology. 2nd ed. Boston: McGraw-Hill; 1999.
3. Darwin C. The Descent of Man. London: John Murray; 1871.
4. Cameron DW, Groves CP. Bones, Stones and Molecules: “Out of Africa and Human Origins”. Burlington: Elsevier Academic Press; 2004.
5. Templeton A. Out of Africa again and again. Nature 2002;416:45-51.doi:10.1038/416045a
6. Templeton AR. Perspective: Genetics and recent human evolution. Evolution 2007;61:1507-19. doi:10.1111/j.1558-5646.2007.00164.x
7. Templeton AR. Gene Flow, Haplotype Patterns and Modern Human Origins. In: Cooper DN, Kehrer-Sawatzki H, eds. Handbook of Human Molecular Evolution. Weinheim, Germany: Wiley-VCH Verlag GmbH & Co.; 2008.
8. Goodman M, Grossman LI, Wildman DE. Moving primate genomics beyond the chimpanzee genome. Trends Genet 2005;9:511-7. doi:10.1016/j.tig.2005.06.012
9. Fouts R, Mills ST. Next of Kin: My Conversations with Chimpanzees. New York: Harper Collins; 1997.
10. Corbey R, Theynissen B, eds. Ape, Man, Apeman: Changing Views Since 1600. Leiden: Department of Prehistory, Leiden University; 1993.
11. Goodall J. The Chimpanzees of Gombe: Patterns of Behavior. Cambridge, Massachusetts: Harvard University Press; 1986.
12. McGrew WC. The Cultured Chimpanzee: Reflections on Cultural Primatology. Cambridge: Cambridge University Press; 2004. doi:10.1017/CBO9780511617355
13. McGrew WC. Chimpanzee Material Culture: Implications for Human Evolution. Cambridge: Cambridge University Press; 1992. doi:10.1017/CBO9780511565519
14. Boesch C, Hohmann G, Marchant LF, eds. Behavioral Diversity in Chimpanzees and Bonobos. New York: Cambridge University Press; 2002.
15. Kano T. The Last Ape: Pygmy Chimpanzee Behavior and Ecology. Stanford: Stanford University

Press; 1992.
16. Wrangham RW. The culture-zone concept becomes untidy. Curr Biol 2006;16:R634-5. doi:10.1016/j.cub.2006.07.031
17. Nishida T, Haraiwa-Hasegawa M, Takahata Y. Group extinction and female transfer in wild chimpanzees in the Mahale mountains. Z Tierpsychol 1985;67:284-301.
18. Nishida T. Local Traditions and Cultural Transmission. In: Smuts BB, Cheney DL, Seyfarth RM, Wrangham RW, Struhsaker TT, eds. Primate Societies. Chicago: University Chicago Press; 1987:462-74.
19. Matsuzawa T. Field Experiments on Use of Stone Tools by Chimpanzees in the Wild. In: Wrangham RW, McGrew WC, Dewaal FBM, Helthe PG, eds. Chimpanzee Cultures. Cambridge: Harvard University Press; 1994.
20. Matsuzawa T. Chimpanzee Mind: 2000–2005, Collection of Articles. Research Report, Ministry of Education, Culture, Sports, Science and Technology, Kyoto, Japan: 2005.
21. Pruetz J. Evidence of cave use by savanna chimpanzees (Pan troglodytes verus) at Fongoli, Senegal: implications for thermoregulatory behavior. Primates 2007;48:316-9. doi:10.1007/s10329-007-0038-1
22. Pruetz J, Bertolani P. Savanna chimpanzees, Pan troglodytes verus, hunt with tools. Curr Biol 2007;17:412-7. doi:10.1016/j.cub.2006.12.042
23. Huffman MA. The C.H.I.M.P.P. Group: A multi-disciplinary investigation into the use of medicinal plants by chimpanzees. Pan Africa News 1994;1:3-5.
24. Whiten A, Goodall J, McGrew WC, et al. Cultures in Chimpanzees. Nature 1999;399:682-5. doi:10.1038/21415
25. Whiten A, Goodall J, McGrew WC, et al. Charting cultural variation in chimpanzees. Behaviour 2001;138:1481-516. doi:10.1163/156853901317367717
26. Savage-Rumbaugh S, Lewin R. Kanzi: The Ape at the Brink of the Human Mind. London: Wiley; 1994.
27. Rumbaugh SS, Shanker S, Taylor TJ. Apes, Language, and the Human Mind. London: Oxford University Press; 1998.
28. Savage-Rumbaugh ES, Fields WM, Segerdahl P, Rumbaugh D. Culture prefigures cognition in Pan/Homo Bonobos. Theoria, International Journal for Theory, History and Foundations of Science 2005;54:311-28.
29. Itani J. Distribution and Adaptation of Chimpanzees in Arid Area. In: Hamburg DA, McCown ER, eds. The Great Apes. Menlo Park: Benjamin-Cummings; 1979:123-35.
30. Wrangham RW, McGrew WC, de Waal FBM, Heltne PG, eds. Chimpanzee Cultures. Cambridge: Harvard University Press; 1994.
31. Morgan BJ, Abwe EE. Chimpanzees use stone hammers in Cameroon. Curr Biol 2006;16:R632-3. doi:10.1016/j.cub.2006.07.045
32. Wrangham RW, Wilson ML. Collective violence: comparisons between youths and chimpanzees. Ann NY Acad Sci 2004;1036:233-56. doi:10.1196/annals.1330.015
33. Wrangham RW, Goodall J. Chimpanzee Use of Medicinal Leaves. In: Heltne PG, Marquardt LA. eds. Understanding Chimpanzees. Cambridge: Harvard University Press; 1989:22-37.
34. Lewin R. Human Evolution: An Illustrated Introduction. 5th ed. Oxford: Blackwell Publishing; 2005.
35. Dart RA. The dentition of Australopithecus africanus. Folia Anatomica Japonica 1934;12:207-22.
36. Cela-Conde CJ, Ayala FJ. Human Evolution: Trails From the Past. Oxford: Oxford University Press; 2007.
37. Nesser R. A Comparison of Statistical and Geometric Reconstruction Techniques: Guidelines for Correcting Fossil Hominin Crania [Masters thesis]. Faculty of Science, University of Cape Town: Rondebosch, South Africa: 2007.
38. Lovejoy CO, Suwa G, Spurlock L, Asfaw B, White TD. The pelvis and femur of Ardipithecus ramidus: The emergence of upright walking. Science 2009;326:71e1-6.
39. Wood B. Hominids. Encyclopedia of Life Sciences. Hoboken: John Wiley & Sons; 2005.
40. Campbell BG, Loy JD, Cruz-Uribe K. Humankind Emerging. Boston: Pearson Education Inc.; 2002.
41. Johanson DC, White TD. A systematic assessment of early African hominids. Science 1979; 203:321-30. doi:10.1126/science.104384

42. Walker A, Leakey R. The Nariokotome Homo erectus Skeleton. Cambridge: Harvard University Press; 1993.
43. Finlayson C. Biogeography and the evolution of the genus Homo. Trends Ecol Evol 2005;20:457. doi:10.1016/j.tree.2005.05.019
44. Wildman D, Uddin M, Liu G, Grossman LI, Goodman M. Implications of natural selection in shaping 99.4% nonsynonymous DNA identity between humans and chimpanzees: enlarging genus Homo. Proc Natl Acad Sci U S A 2003;100:7181-8. doi:10.1073/pnas.1232172100
45. Wildman D, Grossman LI, Goodman M. Functional DNA in Humans and Chimpanzees Shows They are More Similar to Each Other Than Either is to Apes. In: Goodman M, Moffat AS, eds. Probing Human Origins. Cambridge, MA: American Academy of Arts and Sciences; 2002:1-10.
46. Goodman M. Immunochemistry of the primates and primate evolution. Ann NY Acad Sci 1962;102:219-34. doi:10.1111/j.1749-6632.1962.tb13641.x
47. Goodman M. Man's Place in the Phylogeny of the Primates as Reflected in Serum Proteins. In: Washburn S, ed. Classification and Human Evolution. Chicago: Viking Fund Publications in Anthropology; 1963:204-30.
48. Goodman M. Serological analysis of the systematics of recent hominoids. Hum Biol 1963;35:377-436.
49. Uddin M, Wildman DE, Liu G, et al. Sister grouping of chimpanzees and humans as revealed by genomic-wide phylogenetic analysis of brain gene expression profiles. Proc Natl Acad Sci U S A 2004;101:2957-62. doi:10.1073/pnas.0308725100
50. Savage-Rumbaugh ES, Fields WM. Linguistic, Cultural and Cognitive Capacities of Bonobos (Pan paniscus). Culture and Psychology 2000;6:131-53. doi:10.1177/1354067X0062003
51. Savage-Rumbaugh ES, Fields WM, Taglialatela J. Language, speech, tools and writing. A cultural imperative. J Consciousness Studies 2001;8:273-92.
52. Rumbaugh D, Rumbaugh SS. Meaning: A comparative perspective. In: Mandell C, McCabe A, eds. The problem with meaning: Behavioral and cognitive perspectives. Amsterdam: Elsevier Science; 1997:48-180. doi:10.1016/S0166-4115(97)80136-7
53. Rumbaugh DM. Intelligence in Apes and Other Rational Beings. New Haven: Yale University Press; 2003.
54. Enard W, Przeworski M, Fisher SE, et al. Molecular evolution of FOXP2, a gene involved in speech and language. Nature 2002;418:869-72. doi:10.1038/nature01025
55. Jones KA, Porjesz B, Almasy L, et al. Linkage and linkage disequilibrium of evoked EEG oscillations with CHRM2 receptor gene polymorphisms: implications for human brain dynamics and cognition. Int J Psychophysiol 2004;53:75-90. doi:10.1016/j.ijpsycho.2004.02.004
56. Gagneux P, Varki A. Genetic differences between humans and great apes. Mol Phylogenet Evol 2001;18:2-13.doi:10.1006/mpev.2000.0799
57. Caroll SB. Evolution at Two Levels: On Genes and Form. PLoS Biol 2005;3:e245. doi:10.1371/journal.pbio.0030245
58. Yaacov Paz N, Levanon E, Nevo E, et al. A-I RNA editing shows transcriptome diversity in primates (submitted to Proc Natl Acad Sci USA, 2010).
59. Dunker AK, Cortese MS, Romero P, Iakoucheva LM, Uversky VN. Flexible nets. The roles of intrinsic disorder in protein interaction networks. FEBS J 2005;272:5129-48. doi:10.1111/j.1742-4658.2005.04948.x
60. Ronen A. Domestic Fire as Evidence for Language. In: Akazawa T, Aoki K, Bar-Yosef O, eds. Neanderthals and Modern Humans in Western Asia. New York: Plenum Press; 1998.
61. Goren-Inbar N, Sharon G, Melamed Y, Kislev M. Nuts, nut cracking, and pitted stones at Gesher Benot Ya'agov, Israel. Proc Natl Acad Sci U S A 2002;99:2455-60. doi:10.1073/pnas.032570499
62. Cohen J. In the Shadow of Jane Goodall. Science 2010;328:30-5. doi:10.1126/science.328.5974.30
63. Berger LR, de Ruiter DJ, Churchill SE, et al. Australopithecus sediba: A new species of Homo-like australopith from South Africa. Science 2010;328:195-204. doi:10.1126/science. 1184944

Table 1. Australopithecine and *Homo* fossil discoveries.

Hominid species	Shared derived traits
Ardipithecus ramidus, Gona Afar, Ethiopia: 4.4 mya[38]	300–350 cc, facial prognathism, non-obligate bipedal (hallux abduction), arboreal quadruped, decreased canines
Australopithecus anamensis, Kanapoi, Ethiopia: 4.2–3.9 mya[39]	elongated bone shaft, post crania, bipedalism, thick enamel on post-canine dentition
A. afarensis, Afar, Ethiopia: 4.2–2.5 mya[39]	438 cc, derived crania, large post-canine teeth, broad cheek-bones, 105–120 cm tall, non-opposable toes (in Hadar male)[43]
A. garhi, Bouri, Ethiopia: 2.5 mya[39]	Cranial capacity 450 cc, contemporaneous with Olduwai tools
A. africanus, South Africa: 2.5 mya[39]; *A. sediba*: 1.95–1.75 mya[63]	Brain size 454–520 cc, relatively less sexual dimorphism than *A. afarensis*
Paranthropus robustus, South Africa: 2–1 mya[39]	Brain size ~550 cc, contemporaneous with primitive tool use for tuber excavation[43]
P. boisei, East Africa: 2.32–1.41 mya[2]	Relatively flattened face, relative basiocranial mobility, *Homo*-like positioned foramen magnum, brain size 550 cc
Homo habilis, East Africa: 2.5–1.6 mya[2]	Cranial capacity 500–680 cc an increase above the Australopith mean; reduced post-canine dentition; cranium is denser with reduced face size in proportion to skull compared with earlier Hominins, gracility in dentition and crania, reduced prognathism, later *Homo* foot anatomy (specimen identification: ER-1813/ER-1470)
H. rudolfensis, Kenya: 2.5–1.9 mya[2]	crania of 700 cc; increased cranium vault size more spherical, reduced brow ridge, reduced facial projection, wider elongated face, increased molar and premolar enamel thickness yet reduced in size and wider, relatively elongated legs compared to forearms, lighter built lower jaw, increased frontal dentition size, reduced facial size in proportion to cranium/increased basiocranial mobility, enlarged femur at proximal end, post-cranial dentition reduced compared to frontal[43]
H. ergaster, Kenya, East Africa/Georgia, Western Asia (*H. ergaster dmanisi*): 1.9–1.5 mya[2,42]	Increased body-weight, increased elongated legs to shortened forearms ratio, reduced mandible and dentition size, specimen identification: KNM-WT 15000 "Nariokotome3" estimates mean height 170 cm and mean body-weight 58 kg, brain size mean 907 cc; *H. ergaster dmanisi* 650–660 cc, very narrow pelvis exhibits having anatomically modern human (AMH) feature of secondary altricial infants
H. erectus, East Africa, Asia, and Europe: 1.8 mya to 53 kya[42] (thousand years ago)	Cranial bone elongated, continuous projecting brow ridge, dense, flattened forehead; brain size over 870–1,000 cc; AMH-like hyoid; compared with 5 Zhoukoudian femurs from 500,000 years ago body-weight remained the same for 1 million years between *H. ergaster* in Africa and other *H. erectus* (56 kg mean). KNM-WT 15000 shows dentition, height, and cranial capacity also much like the *H. erectus* of China from 500 kya, brain size mean 931 cc; modern human-like facial features apart from being relatively prognathic, anatomically modern human (AMH) barrel-form thorax, ventral invaginate thoracic column, ribs positioned inferior and anteriorly, AMH forearm structure, AMH vertebrae with kyphotic-lordotic curving
H. heidelbergensis, "Broken hill", Zambia/Bodo, Ethiopia; Bringo, Kenya, Africa; Maur, Germany/Sima de Los Huesos, Spain, Europe: 600–240 kya[2]	Cranial capacity 1,300 cc, exceptionally large skull, sagittal keel (an *erectus* feature), double arched Neanderthal-like brow ridge; reduced teeth size and relatively thin mandibular body lacking a chin as in later Neanderthals lacking inner buttressing (BK 67), stands 172 cm, quite robust with the articulation of the elbow increased in size (BK 8518); with substantial increase in skull size, having a more robustly widened face, denser check bones with an exceptionally wide nasal apparatus resembling *H. neanderthalensis* with a prognathic nose, the basiocranium is quite flexible; with extended frontal bone and dense brow ridges (BOD VP-1/1); Maur exhibits reduced ramus and cheek teeth, with big frontal dentition; 180 cm in height taller than *H. Neanderthalensis*; Steinheim female crania exhibits mid-facial prognathism as in Neanderthals yet with a gracile façade yet with thick brow in dual arch form and backward sloping forehead
H. Neanderthalensis, Europe and the	Chignon occipital bun with lambdoidal flatness, having a surface spread out over an unapparent posterior nuchal bulge, the asterion is where

Levant[2] 400-27 kya	the temporal line connects with the lambdoidal symphysis, larger brain capacity than *H. sapiens sapiens*, expanded crania in the parietal and frontal bone areas with an elongated skull, European “classical” Neanderthals are more stockily built, anatomy of robust and shortened limbs and significantly more muscles, adapted sinus morphology to cope with cold, frontal tooth wear assisted in tool use, diastema behind the M_3 (retro-molar space), long pubis bone, denser long bone shafts, narrower medullar cavities, occipital squama convexity and lambdoidal flatness within human variation, Iraq Shanidar exhibits cranial capacity 1,600 cc, elongated vertebral size
H. floresiensis, Flores Indonesia: 90-18 kya	1.06 m; 380 cc; carpals alike *Australopiths/Pan*
H. sapiens sapiens, Herto, Ethiopia: 160 kya; Skhūl/Qafza, Israel: Middle Paleolithic 125–80 kya[2]	Projecting chin, reduced brow ridge, expansion of frontal and parietal bones into a bulbous shape crania, reduction in facial size and prognathism, posterior teeth size reduction with frontal teeth expansion, skeletal gracility, high and straight forehead with a taller and narrower cranium, longer limb length accounts for more efficient mobility, in Skhūl 5: reduced frontal sinuses, well formed chin, large mastoid processes with relatively big lower jaw, straight limb shaft bone, moderately gracile anatomy and brain size 1,518 cc, the face lacks prognathism, eye orbitals are low-positioned and rectangular-shaped and lack of occipital bunning, the cheek-bones form an angled formation (Qafza 6)[2]

Figure 1. Hominin (*Pan*, *Homo*, and *Australopithecus*) phylogeny.

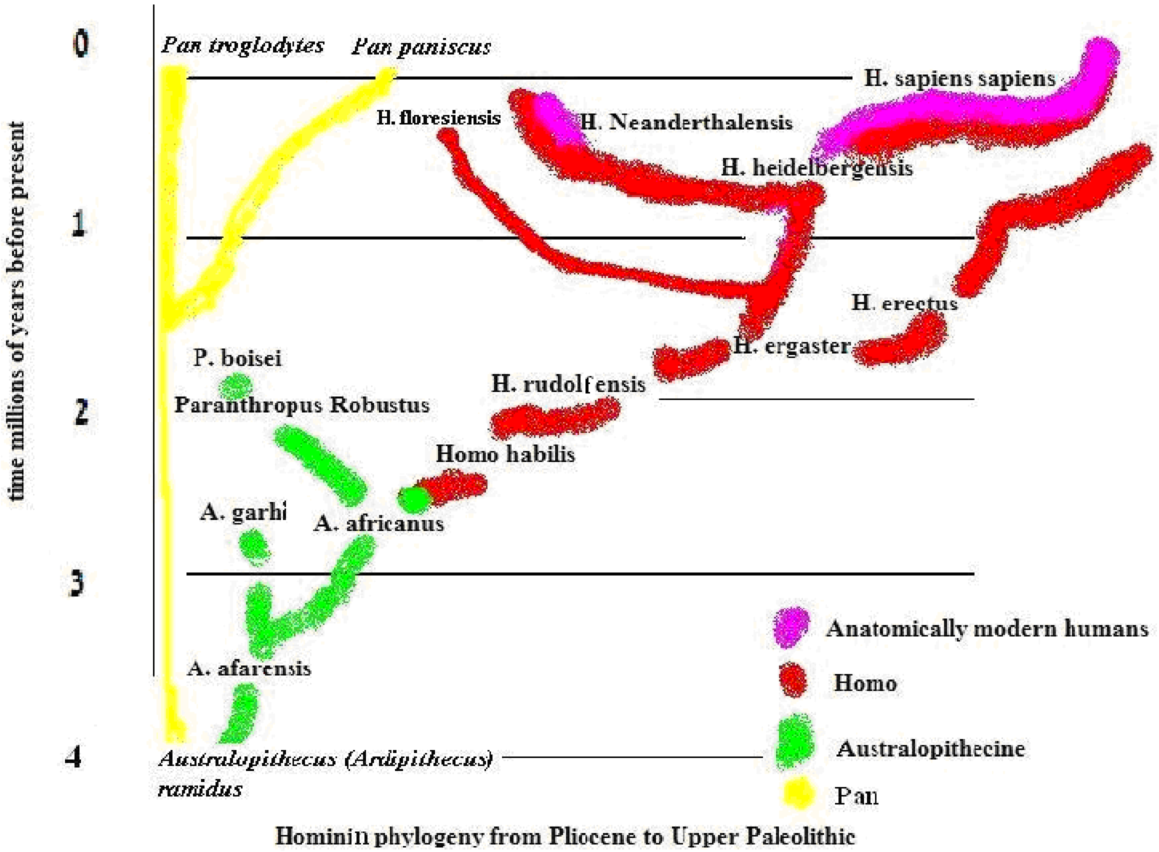

Figure 2. The bonobo Kanzi (*Pan paniscus*) profile.

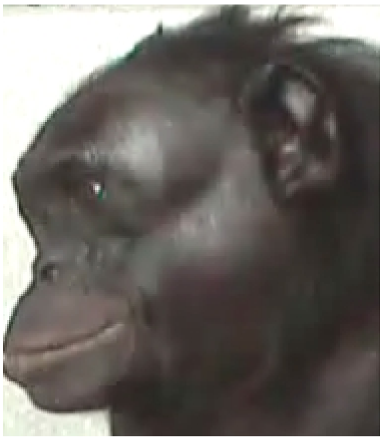

Figure 3. Bonobo Pan-Banisha explaining herself via manual gesture (note joint index finger and thumb along with eye contact) to her guardian, Professor S. Rumbaugh.

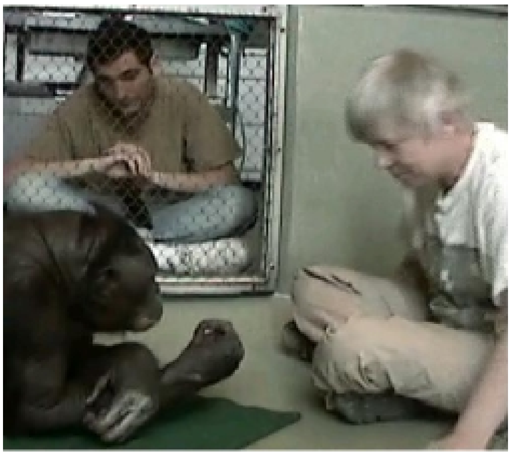

Figure 4. Bonobo Pan-Banisha exhibiting *Australopithecus*/early *Homo* features (head and body proportions).

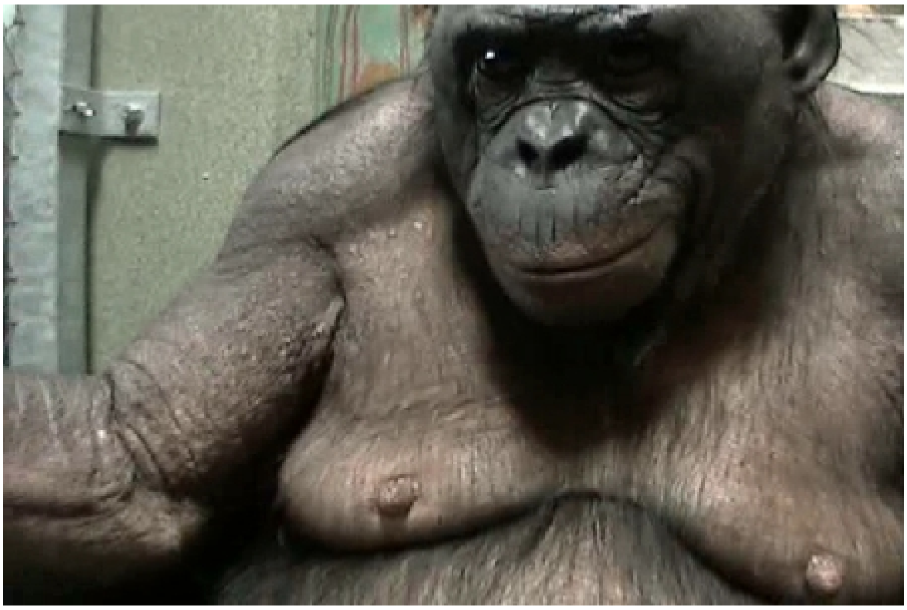

Figure 5. Bonobo Pan-Banisha exhibiting early *Homo*/Australopithecine features

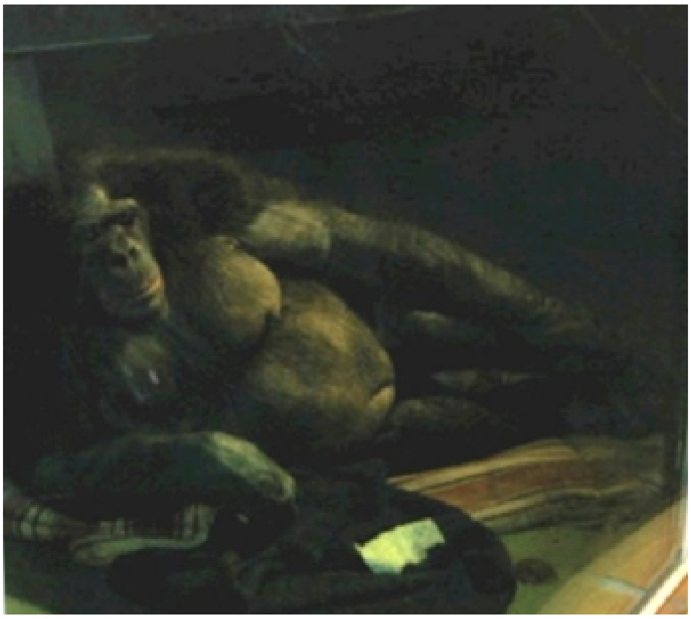

Figure 6. Envisioned illustration of lifetime chimpanzee sanctuary on Mount Carmel, Israel.

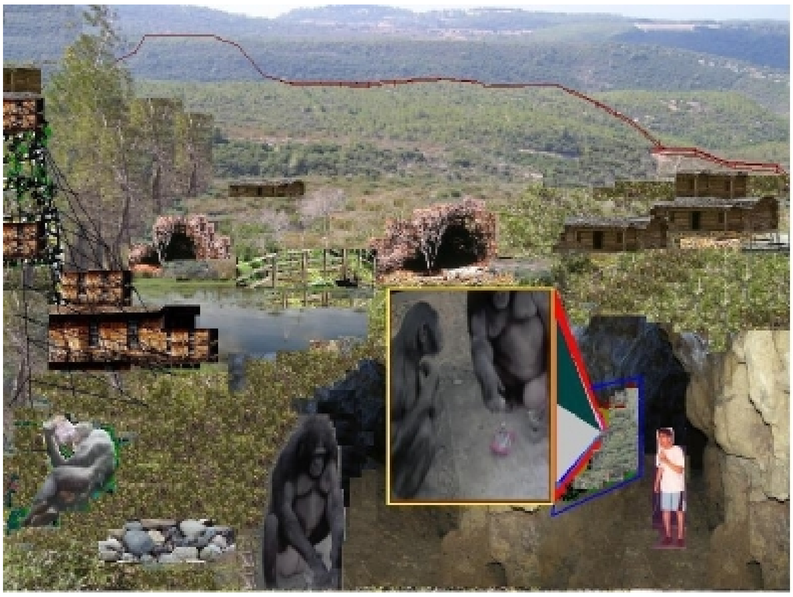

Figure 7. Bonobo Nyota (note the *Homo*-like body features).

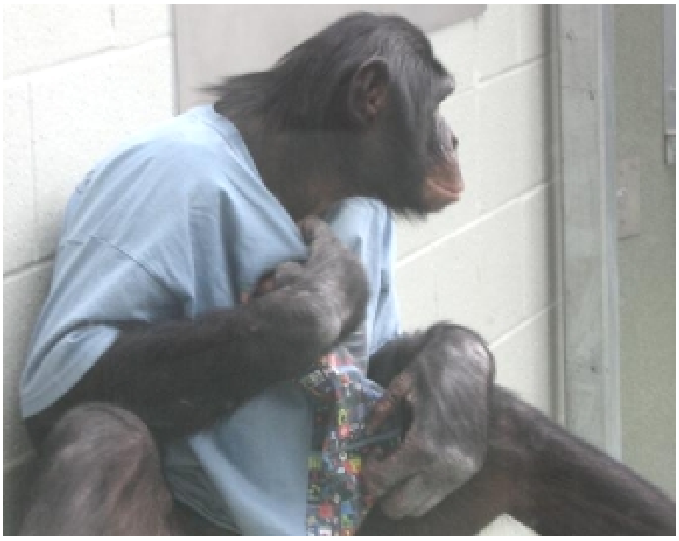

Supplement: Supplementary Material [file e0009_supplement.pdf]
